# Moving research into practice: the diffusion of evidence-based recommendations through professional societies

**DOI:** 10.1186/1748-5908-10-S1-A52

**Published:** 2015-08-20

**Authors:** Elizabeth Neilson, Katherine Clegg Smith, Donald Steinwachs, Darcy F Phelan-Emrick, Robert S Lawrence, Janice V Bowie, Barbara Cohen

**Affiliations:** 1 National Institutes of Health, Office of the Director, Office of Disease Prevention, Bethesda, MD 20892, USA; 2 Johns Hopkins School of Public Health, Baltimore, MD 21205, USA; 3 Collaborative Consultants, Bethesda, MD 20817, USA

## Introduction

There is a substantial need among clinicians for health-related, evidence-based recommendations. Evidence-based recommendations help distill research findings and aid health care providers in making clinical decisions. However, it is infeasible for providers to sort through thousands of available guidelines, and heterogeneity among recommendation developers (e.g., composition, processes, outputs) can make it difficult for clinicians to identify which recommendations are trustworthy, feasible, and applicable to their patient population. Even when there is broad consensus about the quality and utility of recommendations, a range of contextual factors (e.g., the health care system, patient characteristics, enabling resources) can impede implementation. This study examined the diffusion of evidence-based recommendations through professional societies to clinically-trained members, and explored knowledge, attitudes, beliefs, and behaviors regarding evidence-based recommendations and practice. The study had three aims: 1.) Describe the role primary care professional societies play in developing and/or disseminating evidence-based reports and recommendations; 2.) Determine if the needs of primary care providers and their professional societies for evidence-based reports and recommendations are being met; 3.) Describe the value that the federal government contributes to evidence-based practice.

## Methods

To achieve these aims, content analysis was used to examine transcripts from 34 semi-structured telephone interviews of leaders and members from eight health-related professional societies. Nonprobability, purposive sampling of knowledgeable experts enabled in-depth exploration of phenomena. An interview guide was developed using theory-driven concepts and theoretical frameworks, and was pilot tested using cognitive interviewing techniques. The codebook included theory-and data-driven codes and was revised through an iterative process that included intercoder reliability assessments.

## Results

There were differing views on the meaning of "evidence-based", but there was broad agreement on its scientific underpinning and the importance of conducting "evidence-based practice." Professional societies can play several roles (i.e., disseminator, liaison, developer, and/or facilitator) in the promotion of evidence-based recommendations and practice. Views varied on whether the needs of primary care providers and their professional societies for evidence-based reports and recommendations were being met. Federally-sponsored recommendation developers were viewed as valuable contributors to evidence-based practice because of their objectivity, transparency, balance, methodological rigor, and prioritization. Study participants offered many suggestions for improving the development, feasibility, readability, acceptability, and dissemination of evidence-based recommendations. Participants also offered input on how federally-sponsored recommendation developers could strengthen their partnerships with stakeholders, including professional societies and their members.

## Conclusion

The issue of trust was central to participants' attitudes and beliefs; therefore, recommendation developers should integrate transparency and three factors that bolster trust (ability, benevolence, and integrity) into their processes. Federally-sponsored recommendation developers should consider collaborating with professional societies in a variety of ways to develop and disseminate recommendations to facilitate evidence-based practice. The federal government can also promote the use of evidence-based recommendations by improving its guideline clearinghouse, expanding health insurance coverage to more Americans, requiring that recommendations be covered by insurance, and supporting research on point-of-care decision support tools, electronic health records, and workflow training for health providers.

